# Genetics of structural connectivity and information processing in the brain

**DOI:** 10.1007/s00429-016-1194-0

**Published:** 2016-02-06

**Authors:** Sudheer Giddaluru, Thomas Espeseth, Alireza Salami, Lars T. Westlye, Anders Lundquist, Andrea Christoforou, Sven Cichon, Rolf Adolfsson, Vidar M. Steen, Ivar Reinvang, Lars Göran Nilsson, Stéphanie Le Hellard, Lars Nyberg

**Affiliations:** 1 Dr. Einar Martens Research Group for Biological Psychiatry, Center for Medical Genetics and Molecular Medicine, Haukeland University Hospital, 5021 Bergen, Norway; 2 K.G.Jebsen Center for Psychosis Research and the Norwegian Center for Mental Disorders Research (NORMENT), Department of Clinical Science, University of Bergen, 5021 Bergen, Norway; 3 K.G. Jebsen Center for Psychosis Research, Norwegian Center for Mental Disorders Research (NORMENT), Division of Mental Health and Addiction, Oslo University Hospital, 0424 Oslo, Norway; 4 Department of Psychology, University of Oslo, 0317 Oslo, Norway; 5 Umeå Center for Functional Brain Imaging (UFBI), Umeå University, 90187 Umeå, Sweden; 6 Aging Research Center, Karolinska Institutet and Stockholm University, 11330 Stockholm, Sweden; 7 Department of Statistics, USBF, Umeå University, 90187 Umeå, Sweden; 8 Division of Medical Genetics, Department of Biomedicine, University of Basel, 4058 Basel, Switzerland; 9 Institute of Neuroscience and Medicine (INM-1), Research Center Juelich, 52425 Juelich, Germany; 10 Department of Genomics, Life and Brain Center, University of Bonn, 53127 Bonn, Germany; 11 Department of Clinical Sciences, Psychiatry, Umeå University, 90187 Umeå, Sweden; 12 ARC, Karolinska Institutet, Stockholm, Sweden; 13 Department of Radiation Sciences, Umeå University, 90187 Umeå, Sweden; 14 Department of Integrative Medical Biology, Umeå University, 90187 Umeå, Sweden

**Keywords:** Imaging genetics, DTI, GWAS, Processing speed, Fractional anisotropy, Cognition

## Abstract

Understanding the genetic factors underlying brain structural connectivity is a major challenge in imaging genetics. Here, we present results from genome-wide association studies (GWASs) of whole-brain white matter (WM) fractional anisotropy (FA), an index of microstructural coherence measured using diffusion tensor imaging. Data from independent GWASs of 355 Swedish and 250 Norwegian healthy adults were integrated by meta-analysis to enhance power. Complementary GWASs on behavioral data reflecting processing speed, which is related to microstructural properties of WM pathways, were performed and integrated with WM FA results via multimodal analysis to identify shared genetic associations. One locus on chromosome 17 (rs145994492) showed genome-wide significant association with WM FA (meta *P* value = 1.87 × 10^−08^). Suggestive associations (Meta *P* value <1 × 10^−06^) were observed for 12 loci, including one containing *ZFPM2* (lowest meta *P* value = 7.44 × 10^−08^). This locus was also implicated in multimodal analysis of WM FA and processing speed (lowest Fisher *P* value = 8.56 × 10^−07^). *ZFPM2* is relevant in specification of corticothalamic neurons during brain development. Analysis of SNPs associated with processing speed revealed association with a locus that included *SSPO* (lowest meta *P* value = 4.37 × 10^−08^), which has been linked to commissural axon growth. An intergenic SNP (rs183854424) 14 kb downstream of *CSMD1*, which is implicated in schizophrenia, showed suggestive evidence of association in the WM FA meta-analysis (meta *P* value = 1.43 × 10^−07^) and the multimodal analysis (Fisher *P* value = 1 × 10^−07^). These findings provide novel data on the genetics of WM pathways and processing speed, and highlight a role of *ZFPM2* and *CSMD1* in information processing in the brain.

**Electronic supplementary material:**

The online version of this article (doi:10.1007/s00429-016-1194-0) contains supplementary material, which is available to authorized users.

## Introduction

There is a strong genetic influence on brain structure (Thompson et al. 2001, 2014). The genome wide association study (GWAS) approach has proven to be successful in identifying specific genes related to individual differences in cortical and subcortical grey matter volumes (Potkin et al. 2009; Rimol et al. 2010; Bakken et al. 2012; Stein et al. 2012; Hibar et al. 2015). For white matter (WM) pathways that are crucial for speed of information processing (Kail and Salthouse 1994) studies revealed high heritability (Kochunov et al. 2010a, 2016; Jahanshad et al. 2013a). There are conflicting reports on few candidate genes, such as *BDNF* (Chiang et al. 2011), *APOE* (Heise et al. 2011; Westlye et al. 2012; Nyberg and Salami 2014), *ADRB2* (Penke et al. 2010), *GRM3* (Mounce et al. 2014), and *ZNF804A* (Voineskos et al. 2011; Wei et al. 2012; Fernandes et al. 2014) among others. In addition, several groups have undertaken a genome wide approach (Lopez et al. 2012; Jahanshad et al. 2013b; Sprooten et al. 2014). Lopez et al. (2012) used a global measure of white matter tract integrity (*g*_FA_) and identified suggestive evidence for *ADAMTS18*, *LOC388630*. Five SNPs reaching a genome-wide significance were identified in a GWAS of whole brain fractional anisotropy (FA) (Sprooten et al. 2014). This study implicated *GNA13*, *HTR7*, and *CCDC91* genes to influence brain structure and emphasized the role for g-protein signaling in WM development and maintenance. A study by Jahanshad et al. (2013b) identified genome-wide significant association between a variant in the *SPON1* gene and brain connectivity.

Microstructural variation in WM pathways has been linked to measures of information processing speed in both younger adults (Gold et al. 2007; Turken et al. 2008) and in samples of heterogeneous age (Kennedy and Raz 2009; Kochunov et al. 2010b; Penke et al. 2010; Madden et al. 2012; Salami et al. 2012). Increased myelination and axonal diameter is crucial for information processing in the brain (Tessier-Lavigne and Goodman 1996; Haász et al. 2013). Further, same genetic factors mediate the correlation between WM integrity and intellectual performance indicating common physiological mechanism for both (Chiang et al. 2009). The correlation between WM integrity and processing speed although complex (Tuch et al. 2005; Fjell et al. 2011; Tamnes et al. 2012) is consistent not just in healthy subjects but also in patients with psychiatric disorders such as schizophrenia (Karbasforoushan et al. 2015; Wright et al. 2015). This gives a strong rationale to study the potential sources of shared genetic contributions. Notably, recent study on old order amish families and the human connectome project (Kochunov et al. 2016) showed high heritability for both the traits with a high genetic correlation between the two suggesting common genes influencing joint variation in WM microstructure and processing speed. Support for this notion also comes from findings that some genes associated with WM microstructure also associate with processing speed, such as *BDNF* (McAllister et al. 2012), and *APOE* (Luciano et al. 2009). Notably, a recent large scale GWAS study (Ibrahim-Verbaas et al. 2015) on processing speed [letter–digit substitution (LDS)/digit–symbol substitution (DSS) tests] implicated *CADM2, DRD2,* and *PAX3.*

The main purpose of the present study was to identify genes that jointly influence WM microstructural coherence as indexed by whole-brain WM fractional anisotropy (FA) (Pierpaoli and Basser 1996) derived from diffusion tensor imaging (DTI) data and processing speed. FA reflects the directional coherence of water molecules. In WM, diffusion perpendicular to the tract is constrained by the axons and myelin sheaths (Thomason and Thompson 2011), and can thus be used to characterize tissue integrity. In a first step we performed meta-analyses of GWAS data from two independent samples of healthy adults to identify genetic associations with FA and speed tasks (Nilsson et al. 1997, 2004; Espeseth et al. 2012). Thereafter, we tested for genetic associations shared by WM and processing speed, by means of statistical integration of the meta-analysis results.

## Materials and methods

### Participants

The Betula sample examined here was part of a larger prospective cohort study of memory, health and aging (Nilsson et al. 1997, 2004). The current Betula sub-sample consisted of 360 participants (191 females and 169 males) aged between 25 and 80 years (mean = 62.3; SD = 13.3). Of these, 355 had DTI data (188 females and 167 males; age range 25–80 years with mean = 62.3; SD = 13.4). Sample demographics are summarized in Supplementary Table 1. All participants were native speakers of Swedish. The age distribution was skewed towards older participants, with 304 subjects out of 360 between ages 55 and 80 years. None of the participants had any history of severe neurological illness or events; all had normal or corrected to normal vision, and were in good general health. They were non-demented based on an extensive neuropsychological examination and clinical evaluation of data obtained at the test occasions and reviews of medical records starting from adulthood. All participants signed informed consent, in accordance with the guidelines of the Swedish Council for Research in Humanities and Social Sciences.

The Norwegian Cognitive NeuroGenetics (NCNG) sample examined here consisted of 250 participants (166 females and 84 males) aged 18–77 years (mean = 48.8; SD = 16.9) with available DTI data. 220 of these had data available for processing speed (143 females and 77 males, mean age 51.1 ranging from 19 to 77 years). Detailed sample demographics are presented in Supplementary Table 1. The sample was recruited by advertisements in a local newspaper to take part in a larger community-based study on the genetics of cognition (Espeseth et al. 2012). All participants read an information sheet and signed a statement of informed consent approved by the regional committee for Medical and Health Research Ethics (South-East Norway) (Project ID: S-03116). All participants were native speakers of Norwegian and had completed basic education with no history of learning deficits. All participants were interviewed for past or present neurological or psychiatric illnesses known to affect the CNS. Any person with a history of treatment for any of these conditions was excluded from the sample. Furthermore, persons with a depression inventory score indicating undiagnosed depressive illness were excluded. All participants were also interviewed at each visit according to a ‘Life events questionnaire’, which included questions on health, alcohol consumption, smoking habits, physical exercise, and positive and negative life events.

### Genotyping and quality control

Genotyping was performed using commercially available Illumina arrays on DNA isolated from blood samples. The genotyping for both cohorts was performed at the Department of Genomics, Life and Brain Center, University of Bonn, Germany. Betula samples were genotyped using Illumina Omni Express and Omni 1S Bead chip kits. Genotyping and preprocessing was performed using Illumina GenomeStudio software. Manual examination and editing of a subset of the genotype clusters was performed according to the Illumina user guidelines. The following sample and genotyping quality checks were performed using PLINK (Purcell et al. 2007) and GenABEL (Aulchenko et al. 2007) software tools. Samples with call rates <0.97, with high autosomal heterozygosity (FDR < 0.01) or with sex discrepancies were excluded. Since we aimed at a genetically homogeneous sample, the population structure was assessed by multi-dimensional scaling (MDS) analysis using 250 K random SNPs to exclude samples with possible non-Swedish ancestry. Cryptic relatedness was assessed using identity-by-state estimates—IBS (as implemented in GenABEL). The individuals with the higher call rate among the pairs of individuals showing an IBS ≥0.85 were retained. Of the 371 individuals originally genotyped, a total of 10 individuals were excluded: two on the basis of sex discrepancy, four as a result of falling outside the MDS clustering based on the first three components, and four on the basis of cryptic relatedness. This resulted in a data set of 361 individuals. Further, SNPs were filtered and excluded from the analysis if they had a call rate <0.95, minor allele frequency (MAF) <0.01 and Hardy–Weinberg equilibrium (HWE) exact test *P* value <0.001. The final clean data set consisted of 1.4 million SNPs. The same genotyping quality control thresholds were applied to the NCNG sample which was described earlier by Espeseth et al. (2012).

### Genotype imputation

Genotype imputation in the two samples was carried out using the same imputation protocol provided by the enhancing neuroimaging genetics through meta-analysis (ENIGMA), which is accessible at http://enigma.ini.usc.edu/wp-content/uploads/2012/07/ENIGMA2_1KGP_cookbook_v3.pdf. The 1000 Genomes Project Phase I reference haplotype data sets for the European populations (EUR) available at http://www.sph.umich.edu/csg/abecasis/MACH/download/ were used. The protocol used can be summarized as follows. First, using the ChunkChromosome tool (http://genome.sph.umich.edu/wiki/ChunkChromosome), each chromosome was split into manageable pieces of 5000 SNPs, each with an overlap of 500. Each chromosomal chunk was then phased into haplotypes using MaCH (Li et al. 2009, 2010) with 20 rounds and 200 states. The phased haplotypes were then imputed to the reference using minimac (Howie et al. 2012) run for five rounds and 200 states. SNPs with an imputation quality estimate Rsq value >0.5, which is an estimated squared correlation between imputed and true genotypes, are considered to be successfully imputed as recommended by the software developers. The most likely genotypes were then derived from the dosage values, which were rounded to the nearest whole number, and converted to the appropriate genotype. Further quality checks on the genotype files were performed in PLINK to exclude SNPs with a call rate <0.95, minor allele frequency <0.01 and Hardy–Weinberg Equilibrium (exact test) *P* value <0.001. At this stage the SNP overlap between the two samples was assessed and the overlapping SNPs were retained for further analysis. Finally, the following were removed: SNPs with ambiguous alleles between the two samples (i.e. A/T and G/C SNPs), tri allelic SNPs with ambiguous alleles between the two samples, and single base insertions/deletions. This resulted in a final data set of 6.1 million overlapping SNPs between the two samples.

In order to check the accuracy of our genotyping (between the two genotyping experiments) and of the imputation, 11 individuals from the NCNG sample were genotyped along with the Betula sample. Thus these 11 individuals were first genotyped on the Illumina Human610Quad, then run through imputation using the 1000 Genomes reference sample, and finally genotyped on the OmniExpress + Omni1S arrays along with the Betula sample. To calculate the genotyping accuracy between experiments, we compared *N* = 373,105 SNPs that overlapped between the two genotyping experiments. The genotyping reproducibility was 99.97 %. To calculate the imputation accuracy, we compared 666,027 SNPs that had been imputed in these 11 NCNG samples (based on the Illumina Human610Quad genotyping and imputation with the1000 Genomes reference sample) and that were also genotyped with the OmniExpress + Omni1S arrays. The accuracy of the imputation compared to the genotyping was 99.67 %.

### Diffusion MRI protocol and data processing

A detailed description of the DTI MRI methods and subsequent data analysis for the Betula sample is available elsewhere (Salami et al. 2012). In brief, all the MRI data were acquired at Umeå Center for Functional Brain Imaging (UFBI) using the same 3T GE scanner with a 32-channel head coil. Diffusion-weighted data were acquired in three repetitions of 32 independent directions (*b* = 1000 s/mm^2^) and six non-gradient (*b* = 0 s/mm^2^) images. The data matrix was interpolated to a 256 × 256 matrix with an up-sampled spatial resolution of 0.98 × 0.98 × 2 mm. The three runs were then averaged and corrected for head movement and eddy current distortions. The first volume within the averaged volume that did not have a gradient applied was used to generate a binary brain mask. Finally, DTI fit (Behrens et al. 2003) was used to fit a diffusion tensor to each voxel included in the brain mask, yielding a voxel-wise FA volume for each subject.

The data and processing scheme for the NCNG data was performed as previously described (Westlye et al. 2012). Imaging was performed on a 12-channel head coil on a 1.5-T Siemens Avanto scanner (Siemens Medical Solutions, Erlangen, Germany) at Oslo University Hospital, Rikshospitalet. For diffusion weighted imaging a single-shot twice-refocused spin-echo echo planar imaging sequence with the following parameters was used: repetition time (TR)/echo time (TE) = 8590 ms/87 ms, *b* value = 1000 s/mm^2^, voxel size = 2.0 × 2.0 × 2.0 mm, and 64 axial slices. The sequence was repeated twice with 10 *b* = 0 and 60 diffusion-weighted volumes per run. DTI datasets were processed using the FMRIB Software Library (FSL) (Smith et al. 2004). Each volume was affine registered to the first *b* = 0 volume using FMRIB’s linear image registration tool (FLIRT) (Jenkinson et al. 2002) to correct for motion and eddy currents. After removal of non-brain tissue, FA (Basser and Pierpaoli 1996), eigenvectors, and eigenvalue maps were computed by linearly fitting a diffusion tensor to the data.

Both samples were analyzed using the same processing pipeline. FA volumes were transformed into a common space and skeletonized using tract skeleton generation program as employed in tract based spatial statistics (TBSS) (Smith et al. 2006). All volumes were nonlinearly warped to the FMRIB58_FA template by use of local deformation procedures performed by FMRIB’s nonlinear image registration tool (FNIRT) (Andersson et al. 2007). Next, a mean FA volume of all subjects was generated and thinned to create a mean FA skeleton representing the centers of all common tracts. We thresholded and binarized the mean skeleton at FA >0.2. Finally, each subject’s FA map was projected onto the common skeleton, yielding subject-specific FA skeleton maps. Whole-brain FA, computed by averaging FA values across the entire skeleton for each subject, was used in the GWAS.

### Measures of speed of processing

In Betula, a revised version of the Wechsler (1981) DSS test was used: the letter–digit substitution (LDS) test (Nilsson et al. 2005). Briefly, it consists of rows of blank squares, each paired with a letter in a random sequence. A key pairing each letter with a number (1–9) is printed above these rows. Following ten practice trials, participants are asked to fill in the correct number in the blank squares, according to the key, as quickly and accurately as possible. The final test score is the number of correct responses given within a period of 60 s (max score = 125).

In NCNG the digit–symbol substitution (DSS) test from WAIS-R (Wechsler 1981), which has similar basic structure and sensitivity, was used. Briefly, it consists of rows of blank squares, each paired with a number from one to nine in a random sequence. A key pairing each number with a nonsense symbol is printed above these rows. Following seven practice trials, participants are asked to fill in the correct nonsense symbol in the blank squares, according to the key, as quickly and accurately as possible. The final test score is the number of correct responses given within a period of 90 s.

### Correlation between FA and measures of speed of processing

WM FA and speed task measures showed a negative correlation with age in both the samples. For WM FA the correlation with age was *r* = −0.64 and *r* = −0.56, and for the speed task it was *r* = −0.56 and *r* = −0.53 for the Betula and NCNG samples, respectively. The unadjusted correlation between FA and the speed task was *r* = 0.48 in the Betula and *r* = 0.38 in the NCNG sample. When adjusted for age, age^2^ and gender the correlations were modest at *r* = 0.18 in the Betula sample and *r* = 0.11 in the NCNG sample.

### GWAS association testing

We tested for single-marker allelic association under an additive model using linear regression, as implemented in the –linear option in the PLINK software. Age and sex were included as covariates. In addition, the age^2^ term was added to the FA GWAS to account for potential nonlinear relationships between age and WM changes in the brain (Bartzokis et al. 2010; Westlye et al. 2010). Let a SNP has AA, AB and BB as genotypes, and S be the number of B alleles in an individual. Linear regression allows us to include covariates such as gender, age, etc.

$$ {\text{FA}}_{i} = \beta_{0} + \beta_{\text{age}}  {\text{age}}_{i} + \beta_{\text{cal}} C_{i} + \beta_{\text{male}} {\text{male}}_{i} + \beta_{\text{snp}} S_{i} + e_{i} $$

where, $e_{i} \sim N(0, \sigma^{2} )$. We compared the distribution of *P* values obtained under the additive model to that expected under the null hypothesis of no association across the genome and report the quantile–quantile plot to verify the absence of systematic biases due to experimental or other confounding factors such as population stratification. The inflation factor (*λ*) and corresponding standard errors (SE) for the distribution of *P* values were estimated using the estlambda function in the GenABEL software. Manhattan and q–q plots were generated using the tool available at https://github.com/stephenturner/qqman/blob/master/qqman.r.

### Meta-analysis

The overall measure of association in the two samples tested was obtained by meta-analysis, using the inverse variance weighted model from the METAL software package (Willer et al. 2010). The inverse variance based meta-analysis takes inputs: $\beta_{i}$, effect size estimate for study i; ${\text{se}}_{i}$, standard error for study i, with intermediate statistics: $w_{i} = \frac{ 1}{{{\text{se}}_{i}^{2} }}$, ${\text{se}} = \sqrt {1/\mathop \sum \nolimits_{i} w_{i} }$, $\beta = \mathop \sum \nolimits_{i} \beta_{i} w_{i} /\mathop \sum \nolimits_{i} w_{i}$. Overall *Z* score: $Z = \frac{\beta }{\text{se}}$, overall *P* value: $P = 2\phi \left( {\left| { - Z} \right|} \right).$ We further applied stringent filters to the meta-analysis results by only retaining SNPs showing the same direction of effect in both the samples and meta-analysis *P* values (meta *P* values) that were smaller, or more significant, than the two individual *P* values. SNPs with a *P* value below the traditional GWAS threshold (*P* value ≤5 × 10^−8^) were considered genome-wide significant. Additionally, SNPs showing suggestive evidence of association (meta *P* value ≤10^−6^) are also reported in this paper.

Since imputed data were used in this analysis, a high level of linkage disequilibrium (LD) was expected between SNPs showing suggestive evidence of significance. Thus, pair-wise LD for these markers was estimated using—ld option in PLINK, applying the 1000 Genomes Project EUR genotype data release April 2012 as reference and independent loci (as defined by pairwise *r* ^2^ < 0.2 or distance >250 kb) with at least one SNP showing suggestive evidence of significant association. Then, for each of the LD-independent signals, locus-specific plots (Pruim et al. 2010) were generated.

### Genetic overlap test between the traits

To assess the genetic overlap between the two GWAS results we used gene set enrichment analysis (GSEA). GSEA, originally developed for interpreting gene expression studies (Song and Black 2008; Ackermann and Strimmer 2009), is now also applied to GWAS data (Ersland et al. 2012; Fernandes et al. 2013) to test if specific gene sets of interest are enriched for association in a GWAS. First, *P* values from the two meta-analyses were assigned to genes and used to calculate gene-based scores, using the R package LDsnpR (Christoforou et al. 2012) with ENSEMBL66 gene definitions (±10 kb). Since the datasets used were imputed at a high-density level, no additional LD parameters were included. Each ENSEMBL gene was then scored based on the lowest *P* value observed in the gene and corrected for the total number of SNPs in the gene using a modified Sidak’s correction (Saccone et al. 2007). This gene scoring method was found to correlate highly with minimal *P* value permutation-based scoring (Christoforou et al. 2012). Gene set enrichment analysis was performed using the GSEA tool (Mootha et al. 2003; Subramanian et al. 2005) provided for download at http://www.broadinstitute.org/gsea/index.jsp. Given an a priori defined set of genes *S*, the goal of GSEA is to determine whether the members of S are randomly distributed throughout *L* or primarily found at the extremes (top or bottom). An enrichment score (ES) is calculated reflecting the degree of over representation that corresponds to a Kolmogorov–Smirnov-like statistic. Statistical significance (nominal *P* value) of the ES is estimated using an empirical phenotype-based permutation test. When a database of gene sets is evaluated, the estimated significance level is adjusted for multiple hypothesis testing. First, the ES for each gene set is normalized to account for the size of the set and then control the proportion of false positives by calculating the false discovery rate (FDR). The analysis was performed on the pre-ranked list based on the Saccone-corrected gene scores with 1000 permutations. The test parameters were kept at the default settings.

To avoid artifacts in the enrichment test due to regions with high LD, the test sets (i.e., top 50 to top 2000) were pruned to contain only one gene in LD with the same markers (for details, see Fernandes et al. 2013).

The GSEA method used in the present study tests whether the top hits in one trait (top 50, 100, 150, 250, 500, 750, 1000 and 2000 genes) are enriched in the second trait and vice versa. Each GSEA was run three times and a test gene set was considered as significantly enriched only if the nominal *P* value from the GSEA analysis was smaller than 0.05. For each gene set that showed a significant *P* value, random mimic gene sets (*N* = 100) were generated, with each random set having the same number of SNPs and number of genes as the test gene set. GSEA was run for each significant gene set along with its random gene sets and the results were ranked according to the enrichment score (Ersland et al. 2012; Fernandes et al. 2013). The gene set was considered to show truly significant enrichment only if it passed this random gene set test by being in the top 5 % of the ranked list.

### Multimodal analysis

To identify the commonality between the GWAS results from FA and the speed task measure we used a Fisher’s combined probability test for combining the *P* values from the two meta-analyses (Fisher 1932). The idea is that if the ‘k’ null hypotheses are all correct, the *P* values will be uniformly distributed on [0, 1] independently of each other. Then, $X = - 2\mathop \sum \nolimits_{i = 1}^{k} { \ln }(Pi)$ with $X$ following a $X_{2k}^{2}$ from which a *P* value for the global hypothesis can easily be obtained. Since this test does not account for the direction of effect, only those SNPs that showed the same direction of effect in the two meta-analyses were included in the analysis. The results from the combined analysis were also filtered to retain those SNPs that showed a Fisher *P* value that was smaller than the two individual meta *P* values, with each meta *P* value being <0.05.

### Voxel-wise analysis

In keeping with some past reports (e.g., Sprooten et al. 2013), we considered a whole-WM FA-measure in the main analyses. Previous studies suggest that this global measure is a good approximation for relations between specific WM tracts and other variables, such as chronological age (Westlye et al. 2010; Salami et al. 2012). We had no a priori reason to expect this to be different for relations with genes, although some recent data indicate that there may be some tract-specific genetic relations (Kochunov et al. 2015). Based on the top hit from the WM FA analysis (rs149603240 in the *ZFPM2* gene) we conducted some preliminary analyses of general versus local relations between WM and genetic variation by voxel-wise analyses using non-parametric permutation-based statistics estimated via a randomization algorithm implemented in the FSL. Initial data processing and TBSS analysis was performed jointly on both samples up to the point of final statistical analysis, thus ensuring that the analysis was performed using a common mask. The statistical analyses were performed for each sample individually, testing the effect of the relevant allele status on FA while including age and sex as covariates. Ten thousand permutations were run for each contrast (testing positive and negative associations with allele carrier status, respectively), and threshold-free cluster-enhancement (TFCE) (Smith and Nichols 2009) was used for statistical inference to avoid arbitrary initial cluster-forming thresholds. *P* values <0.05 (two-tailed, permutation-based TFCE-corrected) were regarded significant, corrected for multiple comparisons across space. Note that these voxel-wise analyses were only performed for alleles which showed a significant association with mean skeleton FA in both samples to characterize the spatial distribution of the effects, and the voxel-wise correction for family-wise errors should thus be regarded as relatively conservative.

## Results

### GWAS of WM FA

**Fig. 1:**
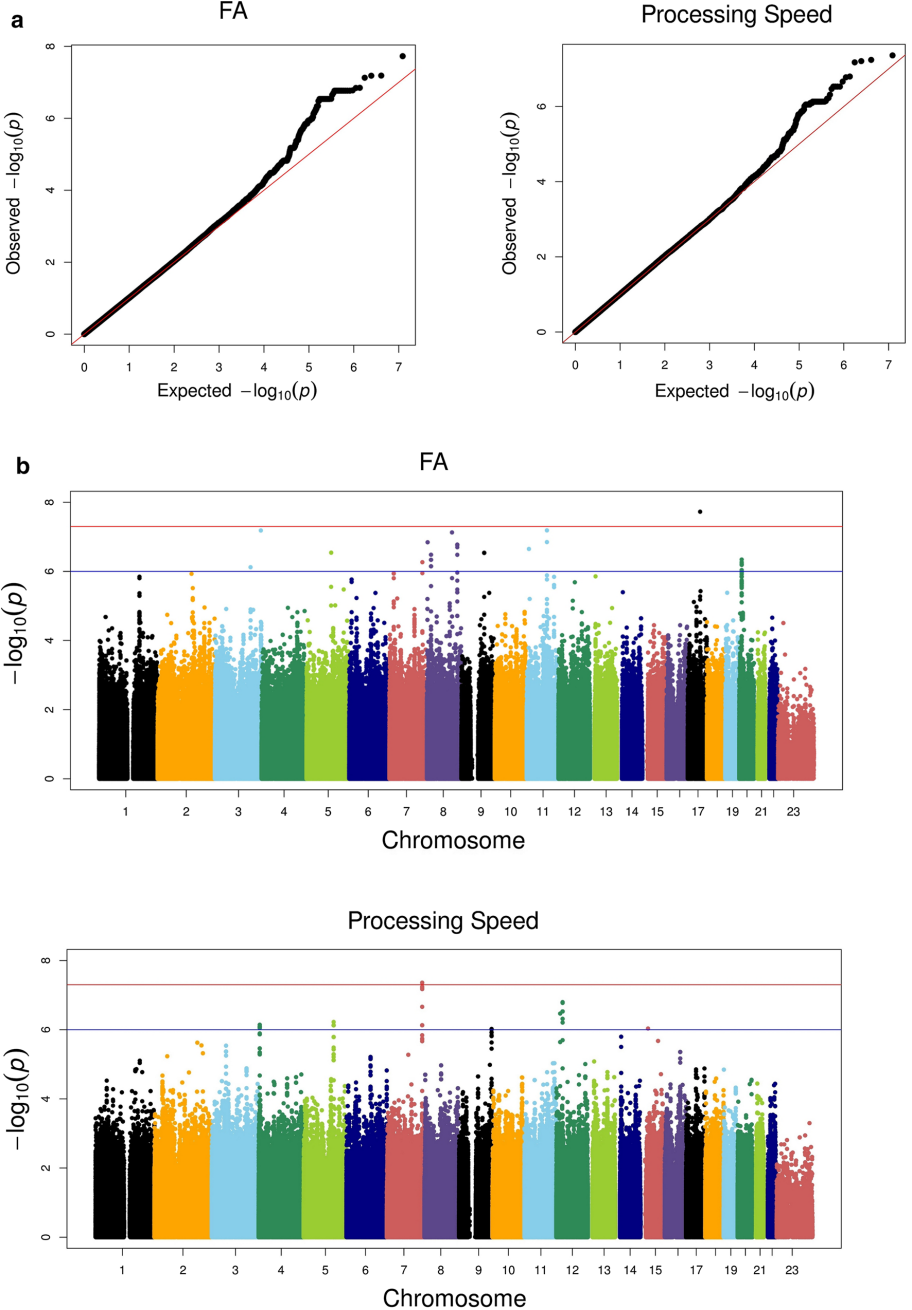
Genome-wide plots for the meta-analysis results of whole-brain skeletal FA and processing speed. **a** q–q plots of meta-analysis results of FA (*λ* = 1.02, SE = 5.42 × 10^−6^), and processing speed (*λ* = 1.01, SE = 3.56 × 10^−6^) with the *diagonal line* representing the expectation under the null hypothesis of no association. **b** Distribution of log-transformed *P* values (Y-axis) from the meta-analysis of FA and processing speed for 6.12 million SNPs tested plotted against the chromosomal positions (X-axis). The *red line* represents the genome-wide significant threshold of 5 × 10^−8^ and the *blue line* represents the threshold for suggestive evidence of 10^−6^

**Fig. 2:**
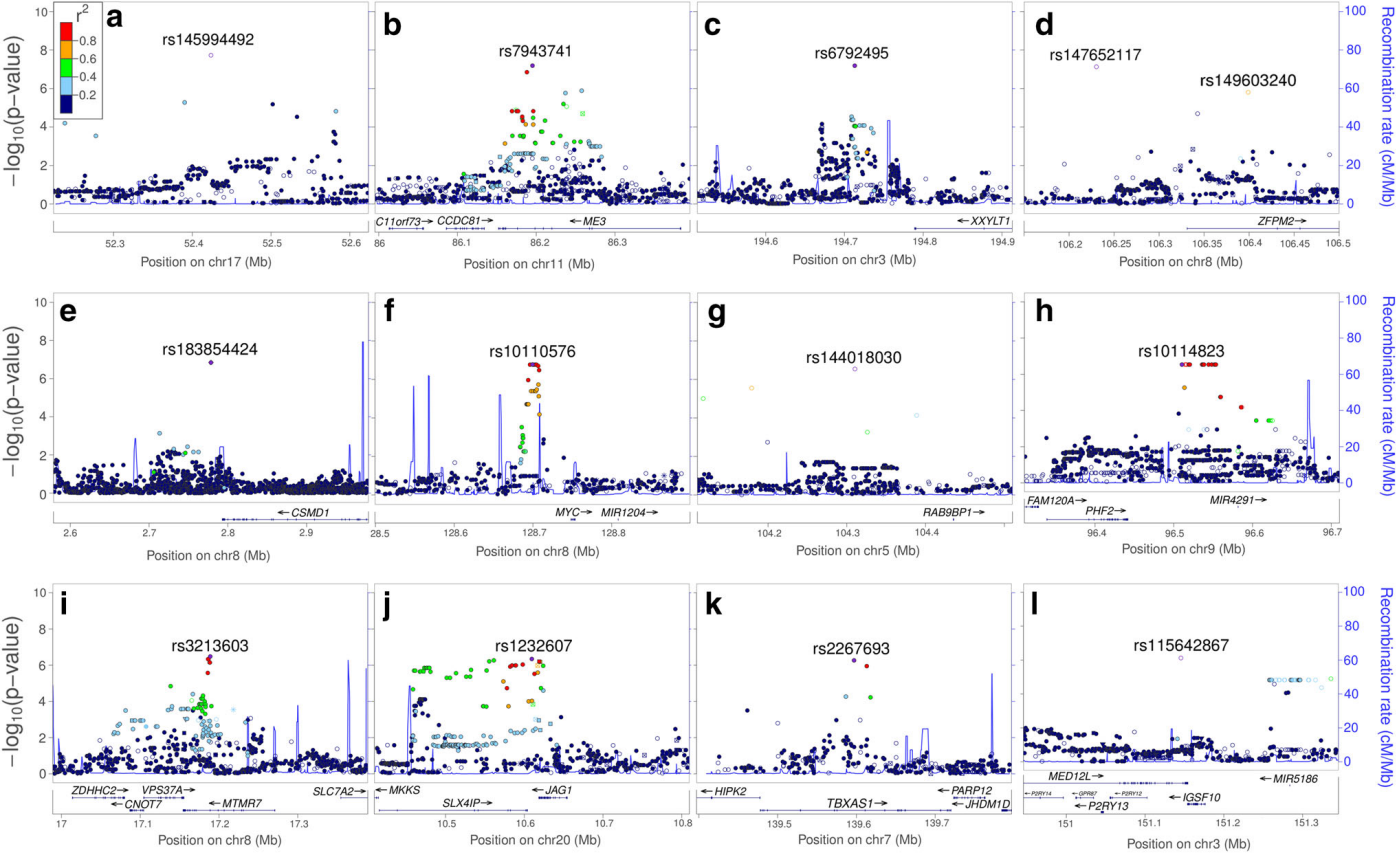
Locus-specific plots highlighting the loci implicated by SNPs reaching a significance threshold of meta *P* value <10^−6^ for FA. **a**–**i** Each plot shows the −log10 *P* value (Y-axis) of SNPs arranged according to their chromosomal positions (X-axis). The locus-specific plots include the genes *ME3* (**b**), *ZFPM2* (**d**), *MTMR7* (**i**), *JAG1* and *SLX4IP*(**j**), *TBXAS1* (**k**), and *IGSF10* and *MED12L* (**l**). The *blue lines* show estimated recombination rates calculated from the HapMap data. The *arrows* represent the genomic locations of genes based on the NCBI Build 37 human assembly. *SNP color* represents LD with the SNP showing highest association in the locus. The SNP annotation is represented as follows: *circles* no annotation; *squares* synonymous or 3′ UTR; *triangles* non-synonymous; *asterisks* TFBScons (in a conserved region predicted to be a transcription factor binding site); *squares* with an X, MCS44 placental (in a region highly conserved in placental mammals)

Using an additive model with age, age^2^ and sex as covariates, associations between 6.1 million SNPs and mean skeletal FA were tested in the two samples followed by meta-analysis. The q–q plot showing the *P* value distribution from the meta-analysis is shown in Fig. 1a (left panel), and the Manhattan plot for the same analysis is presented in Fig. 1b (upper panel). The q–q and Manhattan plots for the individual samples are shown in the Supplementary Figure. One SNP (rs145994492) surpassed the conventional threshold for genome-wide significance of 5 × 10^−8^ in the GWAS of mean skeletal FA. A total of 50 other SNPs showed a meta *P* value ≤10^−6^. Table 1a shows the different genomic loci (12 in total) represented by these SNPs and the most significant SNP(s) in each locus after pruning for LD at *r* ^2^ < 0.8. The SNPs were annotated to the following genes: *ME3*, *MTMR7*, *JAG1*, *SLX4IP*, *TBXAS1*, *IGSF10* and *MED12L* (Fig. 2). One of the top hits, rs147652117, was found to be in strong linkage disequilibrium with an intronic SNP, rs149603240 (pairwise *r* ^2^ = 0.73), in the closest gene *ZFPM2*. The marker rs183854424, which showed suggestive evidence of association, was annotated to the nearest gene, *CSMD1* (14 kb downstream). Several regions with suggestive levels of association were located in intervals that were not near any known genes (intergenic regions; see Table 1). The genes *ZFPM2*, *MTMR7*, and *JAG1* have been implicated in CNS-related functions (Mochizuki and Majerus 2003; Ables et al. 2011; Nielsen et al. 2013). The *CSMD1* gene has been implicated in schizophrenia (Håvik et al. 2011; Ripke et al. 2014) and in neuropsychological deficits in a mouse model (Steen et al. 2013). Further, the GWAS data were mined for polymorphisms implicated in previous reports on candidate genes and recent genome-wide studies of WM FA (Lopez et al. 2012; Jahanshad et al. 2013b; Sprooten et al. 2014) and the findings from two large GWASs of brain volumetric measures (Stein et al. 2012; Hibar et al. 2015) (Supplementary Table 2). None of the genes surpass the suggestive level of significance of meta *P* value <1 × 10^−06^. The lowest *P* value was observed for the gene *ERBB4* (meta *P* value = 0.0005).

**Table 1:** Genome-wide significant and suggestive SNPs from meta-analysis of whole-brain skeleton FA (a), Processing Speed (b), and the multimodality approach (c)

a										
SNP	CHR	BP	LD	A1	A2	Betula (*N* = 355)				
						Geno	Rsq	MAF	Beta	*P*
rs145994492	17	52423691		G	A	Imp	0.89	0.032	–0.0172	3.68 × 10^−06^
rs7943741	11	86195270		G	A	Imp	1.00	0.365	–0.0048	0.00035
rs6792495	3	194713051		C	T	Gen	1.00	0.095	–0.0093	2.98 × 10^−05^
**rs147652117**	**8**	**106230390**		**A**	**G**	**Imp**	**0.91**	**0.020**	**–0.0194**	**4.89** **×** **10** ^**−05**^
rs149603240	8	106398958	0.73	T	C	Imp	0.96	0.025	–0.0153	0.00029
rs183854424	8	2778557		G	T	Imp	0.80	0.076	–0.0104	2.77 × 10^−05^
rs10110576	8	128699454		A	G	Gen	1.00	0.288	0.0042	0.00278
**rs144018030** rs139701334	**5** 5	**104310291** 104517549	0.56	**A** A	**G** C	**Imp** Imp	**0.81** 0.66	**0.014** 0.014	**–0.0146** –0.0146	**0.00927** 0.00927
rs10114823	9	96510399		T	C	Imp	0.99	0.028	–0.0108	0.00692
rs3213603	8	17188911		G	A	Imp	1.00	0.203	–0.0051	0.00094
**rs1232607** rs640336 rs1051421	**20** 20 20	**10609594** 10561369 10620275	0.57 0.6	**T** C T	**C** T C	**Imp** Imp Gen	**1.00** 1.00 1.00	**0.370** 0.256 0.283	**0.0054** 0.0067 0.0064	**8.82** **×** **10** ^**−05**^ 2.29 × 10^−05^ 3.23 × 10^−05^
rs2267693	7	139597043		G	A	Imp	0.99	0.020	–0.0171	0.0003
rs115642867	3	151145469		G	A	Gen	1.00	0.013	–0.0217	0.00022

b										
SNP	CHR	BP	LD	A1	A2	Betula (*N* = 360)				
						Geno	Rsq	MAF	Beta	*P*
**rs6972739**	**7**	**149550942**		**T**	**G**	**Imp**	**0.98**	**0.107**	**–3.363**	**2.23** **×** **10** ^**−06**^
rs3823698	7	149563894	0.66	G	A	Gen	0.93	0.094	–3.508	5.32 × 10^−06^
rs117443760	7	149570724	0.56	C	A	Imp	1.00	0.086	–3.597	5.95 × 10^−06^
rs73168071	7	149541502	0.46	T	C	Imp	1.00	0.165	–2.902	2.87 × 10^−06^
rs16930911	12	26660336		C	T	Imp	1.00	0.031	5.552	1.54 × 10^−05^
rs1467356	12	16338172		G	A	Gen	0.96	0.1859	–2.884	8.65 × 10^−07^
rs73785576	5	126636244		C	T	Gen	1.00	0.060	4.664	5.22 × 10^−06^
rs61642959	4	4961319		T	C	Imp	0.97	0.186	–2.582	5.56 × 10^−06^
rs4779527	15	31736091		T	C	Gen	1.00	0.110	–3.705	9.61 × 10^−07^
rs10776903	9	137689481		A	G	Imp	1.00	0.407	1.89	7.55 × 10^−05^

c							
SNP	CHR	BP	A1	A2	Betula		
					Geno	Rsq	MAF
rs183854424	8	2778557	G	T	Imp	0.80	0.076
rs149603240	8	106398958	T	C	Imp	0.96	0.025
rs74887000	17	41695496	A	G	Gen	0.92	0.034

a									
SNP	NCNG (*N* = 250)					Meta			HGNC Symbol
	Geno	Rsq	MAF	Beta	*P*	Effect	SE	*P*	
rs145994492	Imp	0.54	0.014	–0.0199	0.00213	0.0179	0.0032	1.87 × 10^−08^	Intergenic
rs7943741	Gen	1.00	0.348	–0.0062	6.03 × 10^−05^	0.0054	0.001	6.49 × 10^−08^	*ME3*
rs6792495	Imp	0.64	0.084	–0.0096	0.00089	0.0094	0.0017	6.54 × 10^−08^	Intergenic
**rs147652117** rs149603240	**Imp** Imp	**0.77** 0.73	**0.012** 0.016	**–0.0240** –0.0189	**0.00053** 0.00185	**–0.0209** –0.0165	**0.0039** 0.0034	**7.44** **×** **10** ^**−08**^ 1.57 × 10^−06^	*ZFPM2* *ZFPM2*
rs183854424	Imp	0.58	0.036	–0.0121	0.00197	0.0109	0.0021	1.43 × 10^−07^	Closest gene *CSMD1* (14 kb downstream)
rs10110576	Imp	1.00	0.41	0.0064	1.44 × 10^−05^	0.0053	0.001	1.67 × 10^−07^	Intergenic
**rs144018030** rs139701334	**Imp** Imp	**0.83** 0.66	**0.012** 0.012	**–0.0331** –0.0331	**1.80** **×** **10** ^**−06**^ 1.80 × 10^−06^	**–0.0221** –0.0221	**0.0043** 0.0043	**2.87** **×** **10** ^**−07**^ 2.87 × 10^−07^	Intergenic
rs10114823	Imp	0.97	0.03	–0.0200	6.24 × 10^−06^	–0.015	0.0029	2.91 × 10^−07^	Intergenic
rs3213603	Imp	0.97	0.204	–0.0074	8.87 × 10^−05^	0.006	0.0012	3.29 × 10^−07^	*MTMR7*
**rs1232607** rs640336 rs1051421	**Imp** Imp Gen	**1.00** 0.99 1.00	**0.338** 0.21 0.262	**0.0048** 0.0049 0.0045	**0.00195** 0.00754 0.0058	**0.0051** –0.0059 0.0055	**0.001** 0.0012 0.0011	**4.54** **×** **10** ^**−07**^ 5.42 × 10^−07^ 6.51 × 10^−07^	*JAG1, SLX4IP* *JAG1, SLX4IP* *JAG1, SLX4IP*
rs2267693	Imp	0.96	0.026	–0.0163	0.00069	0.0167	0.0033	5.42 × 10^−07^	*TBXAS1*
rs115642867	Imp	0.81	0.014	–0.0206	0.00131	0.0212	0.0043	7.57 × 10^−07^	*IGSF10, MED12L*

b									
SNP	NCNG (*N* = 220)					Meta			HGNC Symbol
	Geno	Rsq	MAF	Beta	*P*	Effect	SE	*P*	
**rs6972739**	**Imp**	**0.96**	**0.102**	**–4.838**	**0.00692**	**–3.5613**	**0.6504**	**4.37** **×** **10** ^**−08**^	*ZNF862, SSPO, ATP6V0E2*
rs3823698	Gen	1.00	0.096	–5.386	0.00327	3.7885	0.6999	6.21 × 10^−08^	*ZNF862, SSPO, ATP6V0E2*
rs117443760	Imp	0.93	0.096	–5.386	0.00327	3.8785	0.7183	6.69 × 10^−08^	*ZNF862, SSPO, ATP6V0E2*
rs73168071	Imp	0.91	0.148	–3.07	0.04028	–2.9262	0.5645	2.18 × 10^−07^	*ZNF862, SSPO, ATP6V0E2*
rs16930911	Imp	0.96	0.042	7.117	0.003792	–5.8857	1.123	1.60 × 10^−07^	*ITPR2*
rs1467356	Gen	0.98	0.228	–1.602	0.1709	2.6327	0.5163	3.41 × 10^−07^	*SLC15A5*
rs73785576	Imp	1.00	0.036	5.142	0.06126	–4.7212	0.9457	5.97 × 10^−07^	*MEGF10*
rs61642959	Imp	0.98	0.178	–2.34	0.06923	–2.5432	0.5131	7.17 × 10^−07^	Intergenic
rs4779527	Gen	1.00	0.084	–1.434	0.4005	–3.3415	0.6809	9.24 × 10^−07^	*KLF13*
rs10776903	Imp	1.00	0.41	2.846	0.003372	2.076	0.4235	9.49 × 10^−07^	*COL5A1*

c											
SNP	NCNG			Meta FA			Meta speed task			Fisher–P	HGNC symbol
	Geno	Rsq	MAF	Effect	SE	*P*	Effect	SE	*P*		
rs183854424	Imp	0.58	0.036	0.0109	0.0021	1.43 × 10^−07^	1.7805	0.8432	0.03472	1.00 × 10^−07^	Closest gene *CSMD1* (14 kb downstream)
rs149603240	Imp	0.74	0.016	–0.0165	0.0034	1.57 × 10^−06^	–3.0633	1.4161	0.03053	8.56 × 10^−07^	*ZFPM2*
rs74887000	Imp	0.78	0.022	–0.0098	0.003	0.00099	–4.8929	1.207	5.04E−05	8.93 × 10^−07^	Intergenic

A SNP was assigned to a gene if it falls within ±10kbp of the gene based on annotation from ENSEMBL release 66

*CHR* chromosome, *BP* base pair position on the GRC human genome assembly 37, *LD* pair-wise linkage disequilibrium (LD) with the most significant SNP in the close vicinity (bold faced), *A1* first allele for the marker, *A2* second allele for the marker, *Geno* shows whether the SNP has directly been genotyped (Geno) or imputed (Imp), *Rsq* imputation quality estimate *r*-square value, *MAF* minor allele (A1) frequency, *P* *P* value, *effect* overall estimated effect size for A1 in the meta-analysis, *SE* standard error for the overall estimated effect size for A1 in the meta-analysis, *P P* value, *HGNC* *symbol* genes to which the SNPs were assigned

### GWAS of processing speed

**Fig. 3:**
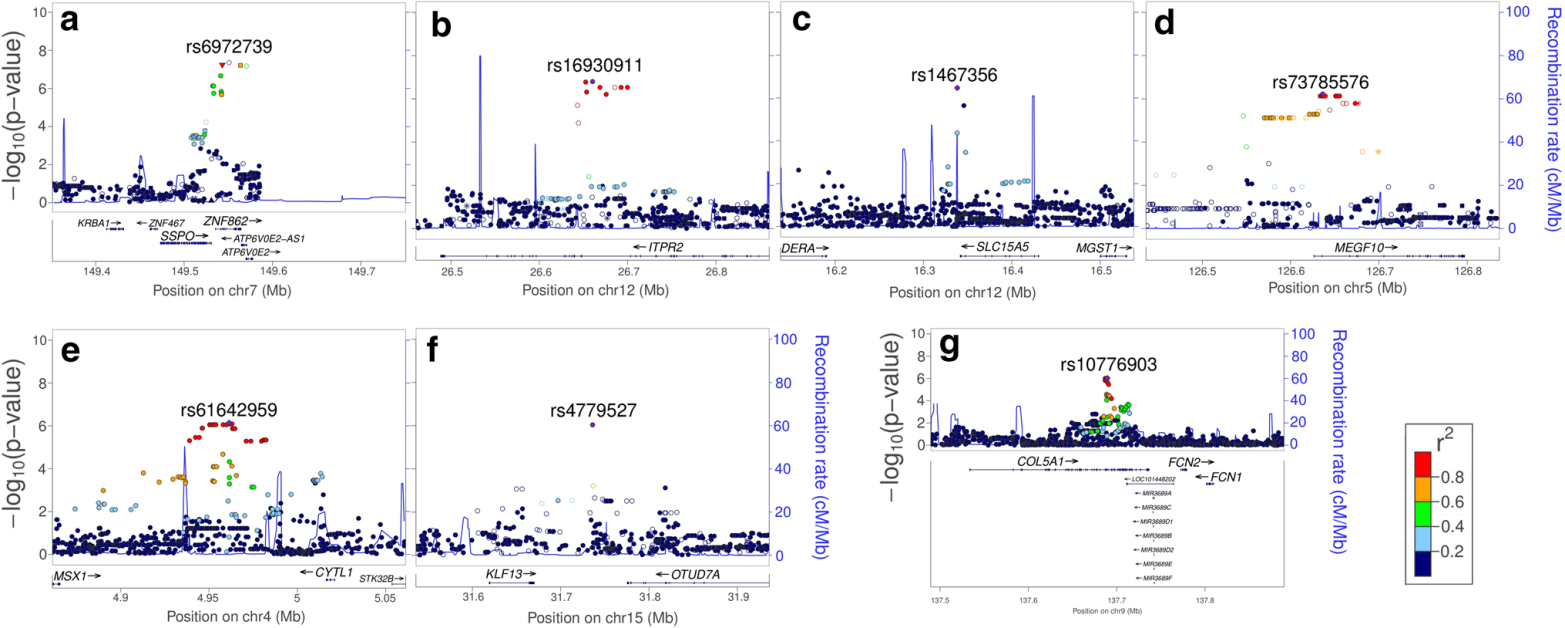
Locus-specific plots highlighting the gene(s) represented by SNPs reaching a genome-wide significance threshold of meta *P* value ≤10^−6^ for processing speed. **a**–**f** Each plot shows the −log10 *P* value (Y-axis) of SNPs (localized in the genic region) arranged according to their chromosomal positions (X-axis). The locus-specific plots include the genes *SSPO*, *ZNF862* and *ATP6V0E2* (**a**), *ITPR2* (**b**), *SLC15A5* (**c**), *MEGF10* (**d**), *KLF13* (**f**), and *COL5A1* (**g**). The *blue lines* show estimated recombination rates calculated from the HapMap data. The *arrows* represent the genomic locations of genes based on the NCBI Build 37 human assembly. SNP color represents LD with the SNP showing highest association in the locus. The SNP annotation is represented as follows: *circles* no annotation; *squares* synonymous or 3′ UTR; *triangles* non-synonymous; *asterisks* TFBScons (in a conserved region predicted to be a transcription factor binding site); *squares* with an X, MCS44 placental (in a region highly conserved in placental mammals)

**Fig. 4:**
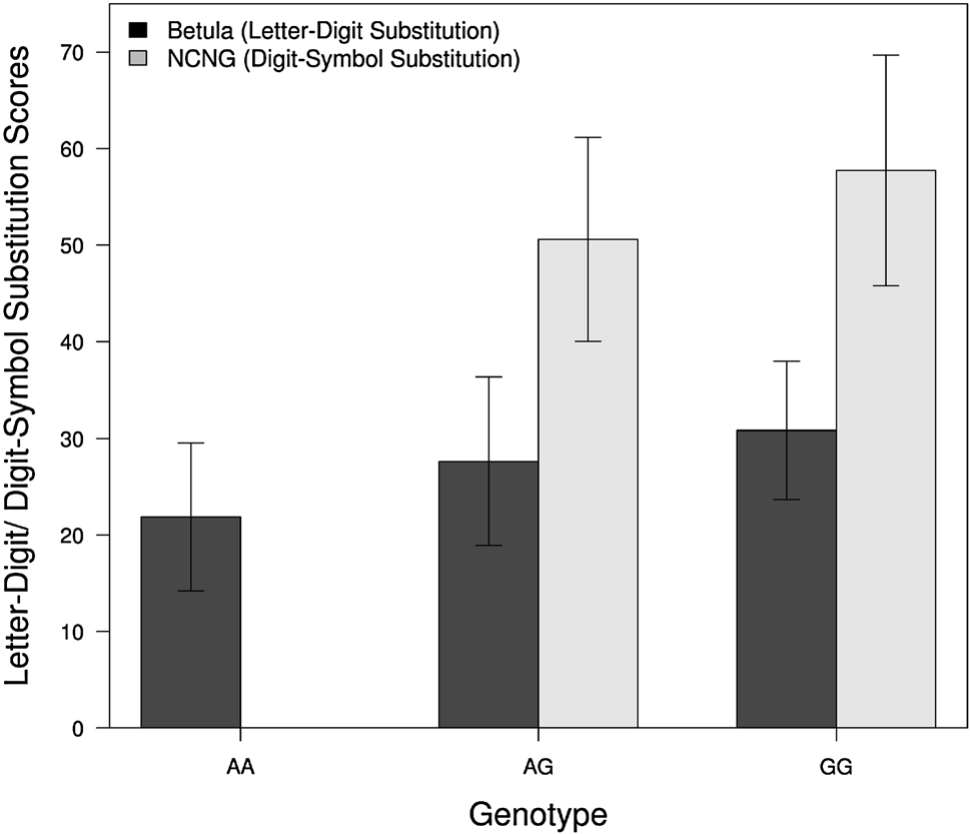
Genotype means for the processing speed task measures in the Betula and NCNG samples for the SNP rs6972739. The *X-axis* shows the three genotypes and the *Y-axis* represents scores from the letter digit substitution and digit symbol substitution tests in Betula and NCNG samples, respectively. *Error bars* indicate one standard deviation from the mean. Number of individuals in each sample that were used to generate the plots: *N* = 360 for Betula and *N* = 220 for NCNG

For processing speed, scores from the speed tasks (letter–digit-/digit–symbol substitution) were used to test for genetic association in the GWAS (Fig. 1a right panel and 1b lower panel). The q–q and Manhattan plots for the individual samples are shown in the Supplementary Figure. Table 1b shows the top hits from the analysis of genes related to speed of processing (meta *P* value ≤10^−6^). Of the top hits, rs6972739 surpassed the conventional threshold for genome-wide significance, and a total of 47 other SNPs showed a meta *P* value ≤10^−6^. Table 1b shows the genomic loci represented by SNPs with meta *P* value ≤ 10^−6^ after LD pruning (*r* ^2^ < 0.8). Together these pruned SNPs represent seven different genomic locations (Fig. 3), which include the genes *SSPO*, *ZNF862*, *ATP6V0E2*, *ITPR2*, *SLC15A5*, *MEGF10*, *KLF13*, and *COL5A1*. Of these, *SSPO*, *ITPR2*, and *MEGF10* have been implicated in CNS-related functions (van Es et al. 2007; Singh et al. 2010; Scheib et al. 2012; Grondona et al. 2012). For illustration, Fig. 4 shows genetic mean differences in performance of the processing speed task in both samples for rs6972739. This SNP is the most strongly associated with processing speed in our meta-analysis and is located in the *SSPO* locus. The GWAS data was mined for polymorphisms implicated in a recent large scale study on processing speed using the LDS/DSS tasks (Ibrahim-Verbaas et al. 2015) (Supplementary Table 2). None of the genes surpass the suggestive level of significance of meta *P* value <1 × 10^−06^. However, nominal significance was observed for *CADM2*, *DRD2*, *PAX3*, and *WDR72* implicated by this study.

### Genetic overlap between two traits: results from GSEA

The top gene sets associated with processing speed were significantly enriched for association in the FA GWAS (Table 2). Random gene set testing validated this finding: these gene sets ranked in the top 5 % when tested along with 100 random genes sets. No significant positive enrichment of top FA hits in processing speed was observed.

**Table 2:** Gene set enrichment analysis of top ranked FA gene sets in processing speed and vice versa

Gene set	FA gene sets in processing speed				Processing speed gene sets in FA			
	Size	NES	NOM *P* value	Validation	Size	NES	NOM *P* value	Validation
Ranked 1–50	47	0.84	0.848	n.d.	49	1.3	*0.017*	At 5 %
Ranked 1–100	96	0.9	0.812	n.d.	98	1.2	*0.041*	At 5 %
Ranked 1–150	143	0.86	0.917	n.d.	148	1.21	*0.015*	At 5 %
Ranked 1–250	241	0.87	0.953	n.d.	246	1.14	*0.027*	At 5 %
Ranked 1–500	486	1	0.511	n.d.	485	1.14	*0.004*	At 1 %
Ranked 1–750	735	1.01	0.441	n.d.	725	1.09	*0.03*	At 5 %
Ranked 1–1000	980	0.98	0.691	n.d.	966	1.09	*0.013*	At 5 %
Ranked 1–2000	1965	0.97	0.828	n.d.	1931	1.04	0.083	n.d.

*Size* number of genes in the set, *NES* normalized enrichment score, *NOM P value* nominal *P* value, *validation* rank when tested along with one hundred random gene sets mimicking the test set in number of genes in the set and number of SNPs in each gene, *n.d*. not determined

Values below significant threshold are in italics

### Identification of shared genetic associations for FA and processing speed

Table 1c shows the three top hits from the analysis of genes related to both FA and speed of processing (*P* value ≤10^−6^). Two of the SNPs identified in the joint analysis, rs183854424 (14 kb downstream of *CSMD1*) and rs149603240 (in an intron of *ZFPM2*) were also among the top hits in the analysis of FA-related genes (Table 1a). The third marker, rs74887000, is an intergenic SNP newly identified in this analysis.

### Spatial distribution of FA effect

**Fig. 5:**
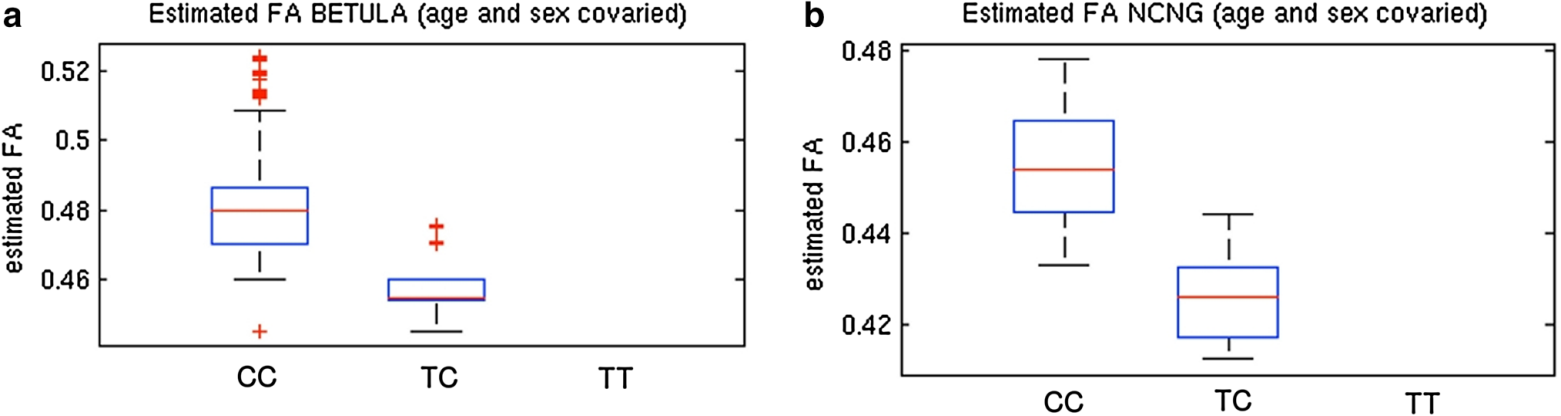
*Box plot* showing the distribution of FA values for the two genotypes observed for the *ZFPM2* SNP (rs149603240). FA values (covaried for age, age^2^ and sex) are plotted on the Y-axis and the two observed genotypes CC and TC on the X-axis. Number of individuals included: *N* = 355 for Betula and *N* = 250 for NCNG

**Fig. 6:**
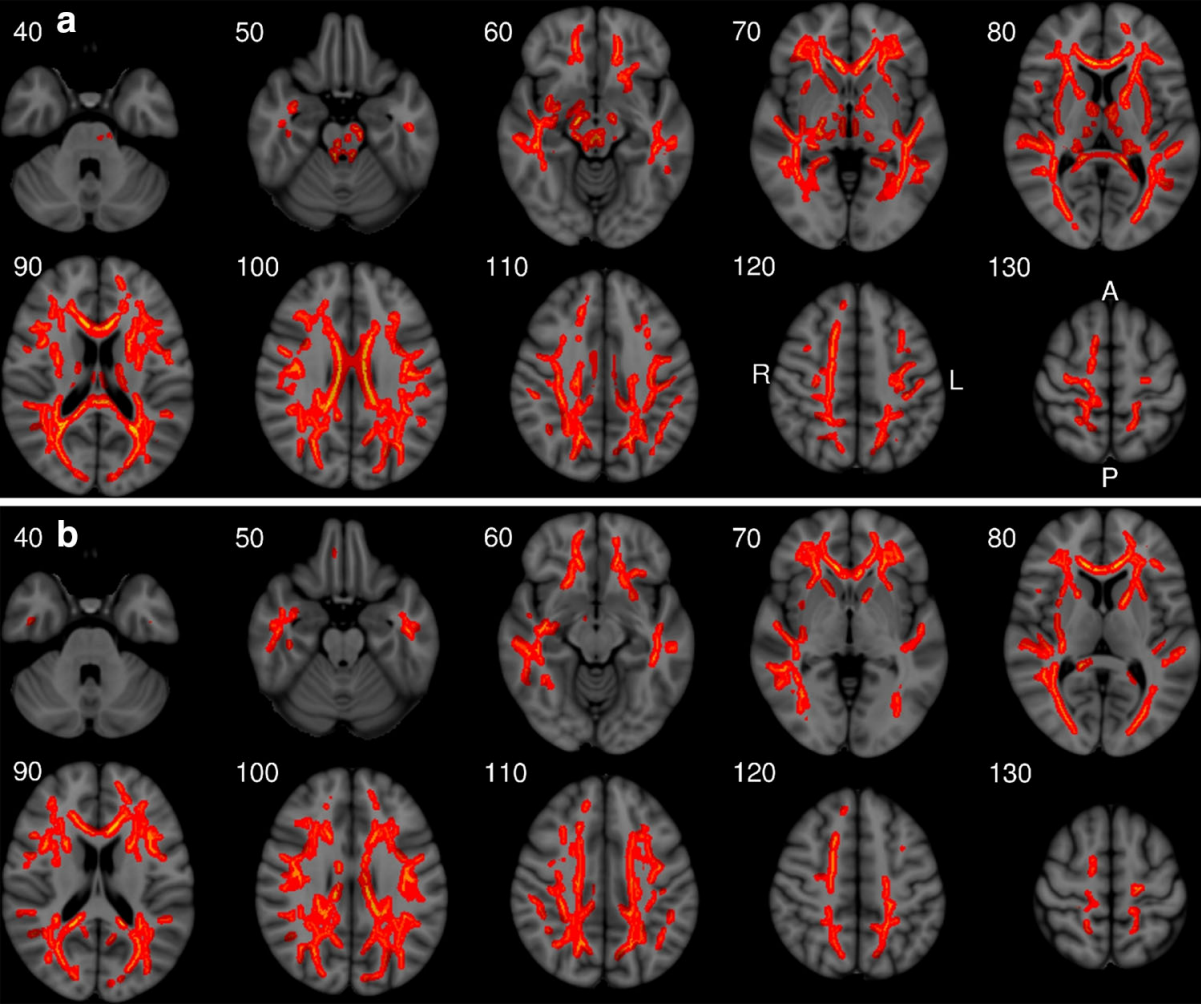
Illustration of voxel-wise analysis of (**a**) Betula and (**b**) NCNG samples using TBSS and permutation-based testing for rs149603240. *Red* denotes voxels with significantly decreased FA in TC carriers compared to CC carriers of rs149603240 (*P* < 0.05, 2-tailed, corrected for multiple comparisons across space using 10 k permutations and TFCE), covarying for main effects of age and sex. Results from tract-based spatial statistics are superimposed on a Montreal Neurological Institute (MNI) common brain template. The numbers refer to the corresponding MNI coordinate for each axial slice. *A* anterior; *P* posterior; *L* left side; *R* right side of the brain

We carried out voxel-wise analyses for the *ZFPM2* intronic SNP rs149603240, which shows a suggestive level of association with WM FA and is significant in the joint analysis with processing speed. A strong effect was observed in both samples, with TC carriers showing decreased FA. Figure 5 depicts the estimated marginal means of global WM FA per allelic group. The effect was anatomically non-specific, covering WM pathways in large parts of the brain (Fig. 6).

## Discussion

This study revealed novel associations between genetic variants in specific loci and mean skeletal FA, a whole-brain index of microstructural coherence. A genome-wide significant association (meta *P* value = 1.87 × 10^−08^) with WM FA was observed for an intergenic SNP (rs145994492), located on chromosome 17. This genetic variant appears to be isolated as no other SNP in the region show suggestive levels of association. Given the low allele frequency (<5 %) of this SNP one should be cautious in the interpretation of this signal. Similarly, several other suggestive association signals were not annotated to genes (intergenic). Genome-wide suggestive significance (meta *P* value ≤10^−6^) was observed for *ME3.* This gene encodes mitochondrial malic enzyme 3, NADP(+) dependent, which catalyzes the oxidative decarboxylation of malate to pyruvate. Interestingly, a copy number variant which includes the *ME3* gene has been associated previously with brain volume (Boutte et al. 2012). The third most significant association in the present study was observed for *ZFPM2* (Zinc Finger Protein, FOG Family Member 2), a cofactor for the GATA family of transcription factors, which regulates the expression of GATA target genes. *ZFPM2* is expressed predominantly in the brain, heart, and testis (Lu et al. 1999) and is relevant in the specification of corticothalamic neurons during neuronal development (Kwan et al. 2008; Nielsen et al. 2013; Deck et al. 2013). Our voxel-wise findings indicate a wide-ranging role of genetic variants in *ZFPM2* in widely distributed WM pathways (Fig. 6). Further, *ZFPM2* is a negative regulator of the PI3K-Akt pathway (Hyun et al. 2009) which in turn is implicated in neurogenesis, neuronal survival, and synaptic plasticity (Spencer 2008), as well as differentiation of oligodendrocytes (Pérez et al. 2013). Other top signals included *MTMR7*, *JAG1*, *SLX4IP*, *TBXAS1*, *IGSF10* and *MED12L*. *JAG1* has been related to many CNS functions such as synaptic plasticity and axon guidance (Ables et al. 2011), which is in accordance with the present findings.

The analysis of genetic variants related to processing speed suggested associations with some candidate genes previously linked to various brain-related phenotypes (*SSPO*, *ITPR2*, *MEGF10*). In humans, the *SSPO* gene encodes the SCP-spondin protein which contributes to commissural axon growth, notably in the posterior commissure (Grondona et al. 2012). The integrity of inter-hemispheric pathways is critical for cognitive information processing speed (Bergendal et al. 2013). The present findings associate the G allele of rs6972739, the most significant SNP in this locus, with faster performance in the processing speed task (Fig. 4). *ITPR2* has been identified as a susceptibility gene in sporadic amyotrophic lateral sclerosis, possibly via its role in glutamate-mediated neurotransmission (van Es et al. 2007).

Although well-designed individual GWA studies like ours have the capacity to identify novel loci, candidate genes and SNPs from previously published GWASs failed to reach nominal significance in our study (Supplementary Table 2). Possible explanations for this inconsistency include limited sample size, polygenic inheritance of complex traits and genetic heterogeneity among different study groups (Liu et al. 2008; Pei et al. 2014).

The primary purpose of the present study was to test the hypothesis that some genes influence both WM microstructure and processing speed. This kind of multi-modal approach (Thompson et al. 2014) has been proposed as a way of enhancing the chances of identifying significant top hits. GSEA between the two traits revealed a significant positive enrichment of the top processing speed genes in the FA GWAS. This implies that the genetic associations identified in the FA GWAS were also enriched for associations relevant in processing speed. The Fisher’s combined *P* value method identified the highest significance (Fisher *P* value = 1 × 10^−07^) for a SNP 14 kb downstream of the *CSMD1* gene, which has been implicated in schizophrenia (Håvik et al. 2011; Ripke et al. 2014). *CSMD1* is also relevant at the functional level, since it has been implicated in neuropsychological deficits in the mouse (Steen et al. 2013). In addition, an intronic SNP in *ZFPM2* was identified in the analysis of shared associations. The *CSMD1* and *ZFPM2* SNPs were both among the top hits in the analysis of FA alone, but were not top hits in the analysis of speed-related genes. Thus, the putative role of the *ZFPM2* or *CSMD1* loci in information processing speed as revealed here would not have been discovered had processing speed been the only phenotype included. As noted above, the *ZFPM2* gene is relevant for the specification of corticothalamic neurons during neuronal development. Corticothalamic neurons are crucial for processing and transmission of sensory information (Alitto and Usrey 2003; Bruno and Sakmann 2006; Briggs and Usrey 2008). A role for *ZFPM2* in mediating processing speed is consistent with these prior observations.

None of the top hits in the processing speed analysis came out as strong hits in the joint analysis. Thus the enrichment effect of adding a phenotype to the multimodal analysis was not symmetrical. This impression is further underscored by results from gene-set enrichment analysis where we observed that the genes associated with processing speed show an enrichment of association in FA, but there is no reciprocal enrichment. We speculate that this asymmetry might be due to the fact that performance in the behavioral task reflects processes other than information processing speed, such as attention and working memory. Another possible explanation is that FA only accounts for a limited amount of the variance in processing speed.

In our study we have successfully identified one genome-wide significant SNP each for WM FA and processing speed, and we have observed multiple loci that show suggestive evidence that the phenotype is affected by many genetic variants (polygenicity). Interestingly, this is consistent with only five novel loci surpassing the genome wide significance in the recent large GWAS (30,177 individuals from 50 different cohorts) of brain subcortical volumetric measures (Hibar et al. 2015). However, our study is limited by several factors, which call for caution in the interpretation. First, it must be noted that even though our sample is among the largest reported for GWAS of FA, the sample size is still rather limited for a GWAS study. It is a general observation in complex trait/disease genetics that common variants (MAF ≥0.01) explain a large proportion of the phenotypic variance with small or modest effects, and large sample sizes are needed for genome-wide significance to be achieved. In addition, the sample composition was biased towards older adults, and this could potentially influence the results, for example if some genes exert a stronger effect at younger ages (but see McClearn et al. 1997 for a suggestion that the same genes contribute to individual differences early as well as later in life). Our study is also limited by the lack of a replication sample. Rather than using one sample as the discovery sample and the second sample as the replication sample, we chose to perform a meta-analysis since this has been shown to be more powerful than to use one sample for discovery and the other for replication (Skol et al. 2006). Although the samples are comparable in terms of phenotypes and genetic origin, there might be sample-specific differences that we would not be able to control for in a mega-analysis, which is why we chose to perform meta-analyses rather than mega-analyses to correct for sample-specific differences. We stress here that the integration of findings from the two samples should be valid as the samples were quite homogeneous from a population perspective, they were genotyped and imputed on the same platform, and the imaging phenotyping and speed tasks were highly comparable. Still, between-site procedural differences did exist, particularly for the imaging procedures. Another point to consider critically is the use of Fisher’s combined *P* value method, which might produce inflated *P* values when the test statistics are correlated. We took measures to counteract this effect, such as matching the directionality of effect and including only those SNPs with meta *P* values < 0.05 prior to testing, and only keeping Fisher *P* values smaller than the test *P* values. For the gene set-based analysis, the method can be prone to bias due to gene set size, gene length, and LD patterns (Wang et al. 2011). To address the issue of variable numbers of SNPs and LD around the markers we applied a modified Sidak’s correction (Saccone et al. 2007) when scoring the genes, which is highly correlated with the gold standard of permutation correction. We pruned our test gene sets to avoid potential intergenic-LD biasing the test statistics. In addition, a non-random distribution of gene size with respect to function has previously been reported; for example, brain-expressed genes are relatively large (Raychaudhuri et al. 2010). This poses a challenge to the gene set-based analysis of GWAS data sets, and we have no control for it in the present study. Another limitation is that WM hyperintensities (WMHIs) are common neuroradiological observations, in particular in samples of older adults, with no obvious clinical or functional implications e.g., (Söderlund et al. 2003). It has been shown that incidental WMHIs might affect FA estimates (Iverson et al. 2011). We did not control for WMHIs in our study, which might be seen as a limitation. Finally, the behavioral and imaging measures have limitations. MRI-derived indices of WM microstructural coherence as indexed by whole-brain FA are indirect measures, so the observed associations could be influenced by additional factors unrelated to or only partly related to brain WM. Relatedly, with the exception of a targeted voxel-wise analysis, we focused on a global index of WM integrity. While there is data to suggest that this is a good proxy for WM status in the brain e.g., (Salami et al. 2012; Sprooten et al. 2013), there is also evidence for heterogeneity among WM tracts in relation to genetic variation (Kochunov et al. 2015). By the latter view, we may well have missed detecting genetic associations that are selective for certain tracts. Similarly, although the letter–digit-/digit–symbol substitution tasks are clearly tapping information processing speed, other cognitive components such as working memory capacity could also influence performance.

In conclusion, the present analyses provide novel data on the genetics of brain WM as well as processing speed, and in particular highlight a key role of *ZFPM2* and *CSMD1* in information processing in the brain. Considering the aforementioned limitations, it will be necessary to validate these findings by analyzing the same phenotypes in further samples.

## Electronic supplementary material

Below is the link to the electronic supplementary material.

Supplementary material 1 (DOCX 46 kb)

Supplementary material 2 (JPEG 1480 kb)

Supplementary material 3 (DOCX 139 kb)
