# Supplementary material for: Genetics of structural connectivity and information processing in the brain
Source: Brain Struct Funct. 2016 Feb 6;221(9):4643–61. doi: 10.1007/s00429-016-1194-0 (PMC5102980; doi:10.1007/s00429-016-1194-0)
Supplement: Supplementary file 1 — Supplementary material 1 (DOCX 46 kb) [file 429_2016_1194_MOESM1_ESM.docx]

**Supplementary Table 1:** Demographics of the samples used for GWAS of FA and speed task measures

|  | FA | | Processing Speed | |
| --- | --- | --- | --- | --- |
|  | Betula | NCNG | Betula  (Letter Digit Substitution) | NCNG  (Digit Symbol  Substitution) |
| Sample size | 355 | 250 | 360 | 220 |
| Age Range (years) | 25 - 80 | 18 - 77 | 25 - 80 | 19 - 77 |
| Age (years) | 62.3±13.4 | 48.8±16.9 | 62.3±13.3 | 51.1±15.6 |
| Sex (# Women) | 188 | 166 | 191 | 143 |
