# Supplementary figures and images for: Genetics of structural connectivity and information processing in the brain
Source: Brain Struct Funct. 2016 Feb 6;221(9):4643–61. doi: 10.1007/s00429-016-1194-0 (PMC5102980; doi:10.1007/s00429-016-1194-0)

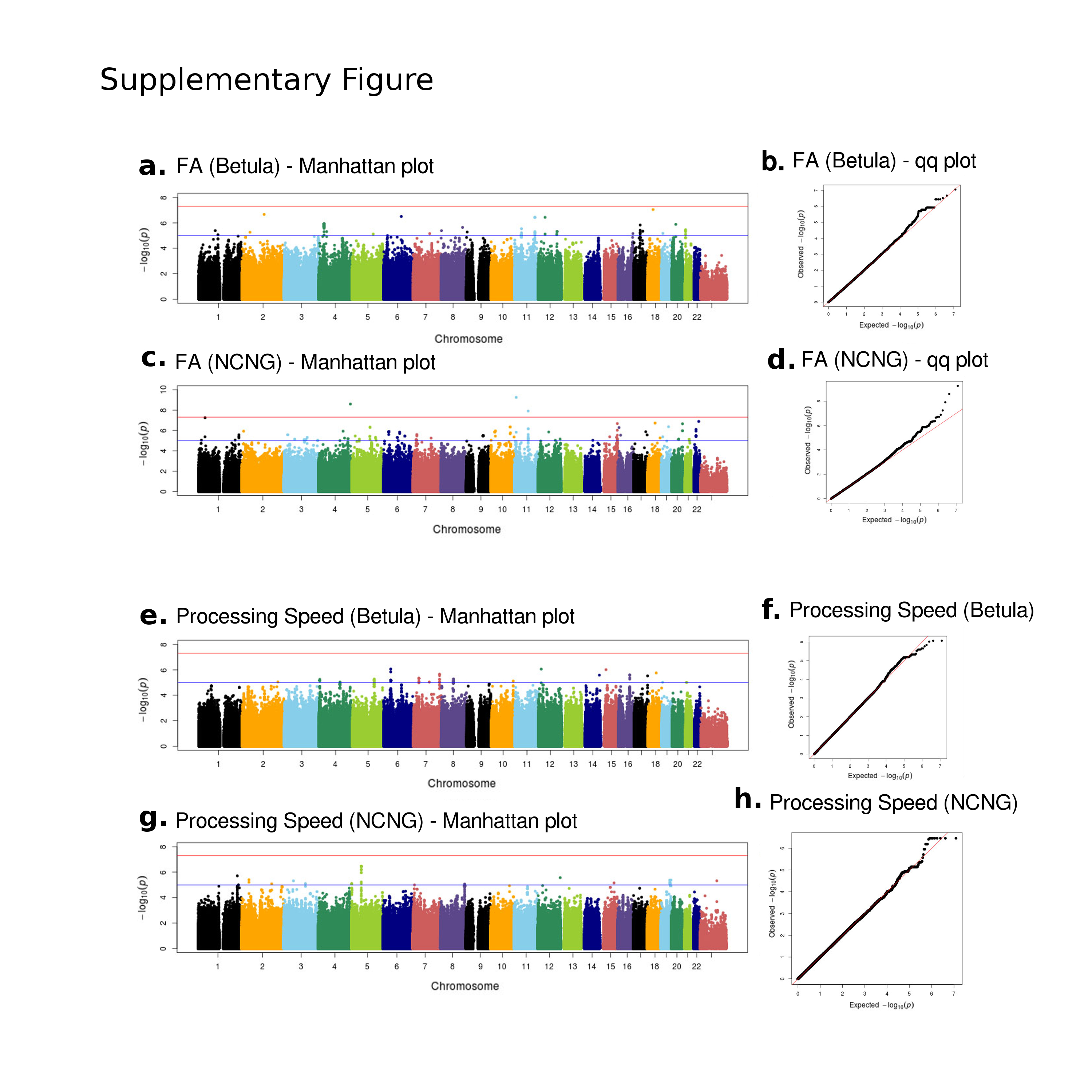

Supplement: Supplementary file 2 — Supplementary material 2 (JPEG 1480 kb) [file 429_2016_1194_MOESM2_ESM.jpg]
