# Supplementary material for: Genetics of structural connectivity and information processing in the brain
Source: Brain Struct Funct. 2016 Feb 6;221(9):4643–61. doi: 10.1007/s00429-016-1194-0 (PMC5102980; doi:10.1007/s00429-016-1194-0)
Supplement: Supplementary file 3 — Supplementary material 3 (DOCX 139 kb) [file 429_2016_1194_MOESM3_ESM.docx]

**Supplementary Table 2**:

**a)** Association results of WM FA GWAS for candidate genes and genes/SNPs identified in previous GWAS studies in healthy individuals.

**SNPs reported in previous GWAS Studies**:

| **Reference** | **SNP** | **Chromosome** | **Gene** | **MAF Reported** | **Effect Reported** | **P-value Reported** | **A1** | **A2** | **MAF** | **Beta** | **P-value** | **SNP showing lowest P-value** | **Lowest P-value observed in the gene** | **LD observed between the reported and the SNP showing lowest P-value r-sq (D-prime)** |
| --- | --- | --- | --- | --- | --- | --- | --- | --- | --- | --- | --- | --- | --- | --- |
| Sprooten et al., 2014 | rs10853057 | 17 | GNA13 | 0.23 | 39.56 (chi-sq) | 3.18×10−10 | C | T | 0.08 | 0.0023 | 0.2315 | rs2011307 | 0.0271 | 0.406 (0.84) |
|  | rs12249377 | 10 | HTR7 | 0.31 | 39.51 (chi-sq) | 3.26×10−10 | A | C | 0.17 | -0.0013 | 0.3436 | rs74595112 | 0.004715 | 0.003 (1) |
|  | rs258415 | 12 | Intergenic | 0.3 | 37.77 (chi-sq) | 7.97×10−10 | C | A | 0.15 | 0.0023 | 0.0874 | - | - |  |
|  | rs1991867 | 16 | Intergenic | 0.4 | 36.03 (chi-sq) | 1.94×10−9 | C | T | 0.15 | 0.0007 | 0.6106 | - | - |  |
|  | rs1361277 | 1 | Intergenic | 0.29 | 31.72 (chi-sq) | 2×10−8 | C | T | 0.18 | 0.0026 | 0.0331 | - | - |  |
|  |  |  |  |  |  |  |  |  |  |  |  |  |  |  |
| Jahanshad et al., 2013 | rs2697846 | 11 | SPON1 | 0.38 | 0.0018 (Beta) | 2.22×10−9 | T | G | 0.38 | 0.0006 | 0.5299 | rs11023156 | 0.0076 | 0.001 (0.071) |
|  | rs2618516 | 11 | SPON1 | 0.36 | 0.0018 (Beta) | 5.82×10−10 | T | C | 0.33 | 0.0003 | 0.7588 | - | - | 0 (0.046) |
|  | rs10832160 | 11 | SPON1 | 0.34 | 0.0017 (Beta) | 7.58×10−9 | G | A | 0.34 | -0.0007 | 0.489 | - | - | 0.002 (0.103) |
|  | rs11023052 | 11 | SPON1 | 0.34 | 0.0018 (Beta) | 1.07×10−9 | T | C | 0.34 | 0.0008 | 0.4281 | - | - | 0.001 (0.097) |
|  | rs7124311 | 11 | SPON1 | 0.33 | 0.0017 (Beta) | 7.02×10−9 | G | T | 0.34 | -0.0007 | 0.5052 | - | - | 0.001 (0.083) |
|  |  |  |  |  |  |  |  |  |  |  |  |  |  |  |
| Lopez et al., 2012 | rs7192208 | 16 | ADAMTS18 | 0.11 | -0.48 (Beta) | 1.65×10−6 | G | T |  | -0.0021 | 0.2518 | rs274517 | 0.0083 | 0.001 (0.066) |
|  | rs946836 | 1 | LOC388630 | 0.33 | 0.3 (Beta) | 5.08×10−6 | T | C |  | -0.0008 | 0.4594 | - | - |  |

**ENIGMA2 subcortical brain structures** (Hibar et al., 2015):

| **Test/Phenotype** | **SNP** | **Chromosome** | **A1** | **A2** | **MAF** | **Beta** | **P-value** |
| --- | --- | --- | --- | --- | --- | --- | --- |
| Putamen | rs945270 | 14 | T | C | 0.3931 | -0.0012 | 0.549 (rs12587289 closest SNP) |
| Putamen | rs62097986 | 18 | A | C | 0.3865 | 0.0008 | 0.4097 |
| Putamen | rs6087771 | 20 | T | C | 0.3259 | -0.0011 | 0.3452 |
| Putamen | rs683250 | 11 | A | G | 0.3377 | 0.0009 | 0.374 |
| Caudate | rs1318862 | 11 | T | C | 0.3931 | 0.0003 | 0.7999 |
| Hip. | rs77956314 | 12 | T | C | 0.0752 | 0.0004 | 0.821 |
| Hip. | rs61921502 | 12 | T | G | 0.1425 | -0.0023 | 0.09588 |
| ICV | rs17689882 | 17 | A | G | 0.2296 | 0.0001 | 0.9267 |

**ENIGMA subcortical brain structure**s (Stein et al., 2012):

| **Test/Phenotype** | **SNP** | **Chromosome** | **A1** | **A2** | **MAF** | **Beta** | **P-value** |
| --- | --- | --- | --- | --- | --- | --- | --- |
| Mean bilateral hippocampal volume | rs7294919 | 12 | T | C | 0.08179 | -0.0001 | 0.9337 |
| Intracranial volume | rs10784502 | 12 | T | C | 0.4749 | 0.0003 | 0.7558 |
| Total brain volume | rs10494373 | 1 | A | C | 0.07256 | 0.0011 | 0.534 |

**Candidate Genes**:

| **Reference** | **SNP** | **Gene** | **Chromosome** | **MAF** | **Minor Allele** | **Major Allele** | **Beta** | **P-value** | **SNP showing lowest P-value** | **P-value** | **LD observed between the reported and the SNP showing lowest P-value r-sq (D-prime)** |
| --- | --- | --- | --- | --- | --- | --- | --- | --- | --- | --- | --- |
| Thomason et al., 2010 | rs4680 (Val158Met) | *COMT* | 22 | 0.4842 | A | G | 0.0003 | 0.7962 | rs165599 | 0.0016 | 0.185 (0.624) |
| Tost et al., 2013 | rs6265 (Val66Met) | *BDNF* | 11 | 0.2032 | A | G | -0.0012 | 0.3456 | rs189740576 | 0.0047 | 0.035 (1) |
| Sprooten et al., 2011 | rs821616 (Ser704Cys) | *DISC1* | 1 | - | - | - | - | - | rs1417584 | 0.0065 | 0.005 (0.098) |
| Fernandes et al., 2014 | rs1344706 | *ZNF804A* | 2 | 0.3931 | T | G | -0.0002 | 0.8554 | rs7580993 | 0.0325 | 0.046 (1) |
| Wang et al., 2015 | rs7412 | *APOE* | 19 | 0.06596 | T | C | -0.0006 | 0.7427 | rs429358 | 0.2739 | 0.005 (0.679) |
|  | rs429358 |  |  | 0.1425 | T | C | 0.0016 | 0.2655 | - | - | - |
|  | rs4420638 |  |  | 0.186 | A | G | 0.0013 | 0.3519 | - | - | 0.659 (0.952) |
| Penke et al., 2010 | rs1042713 | *ADRB2* | 5 | 0.3826 | A | G | 0.0013 | 0.2168 | rs1042720 | 0.2996 | 0.017 (0.24) |
|  | rs1042714 |  |  | - | - | - | - | - | - | - | 0.173 (0.738) |
| Mounce et al., 2014 | rs7808623 | *GRM3* | 7 | 0.07388 | T | G | -0.0018 | 0.3494 | rs117460381 | 0.0222 | 0.001 (0.063) |
| Konrad et al., 2009 | rs839523 | *ERBB4* | 2 | 0.2982 | A | G | 0.0009 | 0.425 | rs16847874 | 0.0005 | 0.003 (0.453) |
| Braskie et al., 2011 | rs11136000 | *CLU* | 8 | 0.3945 | T | C | 0.0003 | 0.7992 | rs9331934 | 0.3086 | 0.025 (1) |
| Nickl-Jockschat et al., 2014 | rs3918342 | *DAOA* | 13 | 0.496 | T | C | 0.0023 | 0.02253 | rs1557072 | 0.0096 | 0.001 (0.216) |
|  | rs1421292 |  |  | - | - | - | - | - | - | - | - |
| Jonassen et al., 2012 | Microsatellite repeat | *SLC6A4* | 17 | - | - | - | - | - | rs11872020 | 0.0607 | - |
| Shen et al., 2010 | rs2075650 | *TOMM40* | 19 | 0.1266 | A | G | 0.0019 | 0.1614 | rs405697 | 0.0862 | 0.054 (1) |
| Braskie et al., 2012 | rs4661063 | *NTRK1* | 1 | 0.08311 | A | G | 0.0005 | 0.754 | rs6339 | 0.0701 | 0.059 (0.354) |
| Braskie et al., 2013 | rs1017412 | *NTRK3* | 15 | 0.2982 | A | G | 0.001 | 0.3832 | rs62022271 | 0.01 | 0.008 (1) |
|  | rs2114252 |  |  | 0.2454 | A | C | 0 | 0.991 | - | - | - |
|  | rs16941261 |  |  | - | - | - | - | - | - | - | - |
|  | rs3784406 |  |  | 0.4248 | T | C | 0.001 | 0.307 | - | - | - |
|  | rs7176429 |  |  | 0.3285 | T | G | -0.0009 | 0.3897 | - | - | - |

**b)** Association results of processing speed GWAS findings and candidate genes implicated in processing speed in the study by **CHARGE consortium** (Ibrahim-Verbaas, et al., 2015):

| **Test/Phenotype** | **Gene** | **SNP** | **Chr** | **A1** | **A2** | **MAF** | **Effect Reported** | **P-value Reported** | **Beta** | **P-value** | **SNP showing lowest P-value in the processing speed GWAS** | **P-value** | **LD observed between the reported and the SNP showing lowest P-value r^2^ (D')** |
| --- | --- | --- | --- | --- | --- | --- | --- | --- | --- | --- | --- | --- | --- |
| LDST/DSST (GWAS finding) | *CADM2* | rs17518584 | 3 | T | C | 0.36 | 5.43 | 5.56×10−8 | 0.3189 | 0.4764 | rs114906451 | 0.01485 | 0.001 (0.302) |
| LDST/DSST (GWAS finding) | *DRD2* (11q.25) | rs2734839 | 11 | T | C | 0.39 | 5.123 | 2.95×10−7 | -0.6938 | 0.1212 | rs77264605 | 0.003807 | 0.007 (0.283) |
| LDST/DSST (GWAS finding) | *PAX3* | rs2118666 | 2 | T | G | 0.46 | -4.95 | 7.45×10−7 | 0.2661 | 0.5383 | rs12616136 | 0.03667 | 0.002 (0.107) |
| LDST/DSST (from previous reports) | *APOE-E4* | rs4420638 | 19 | G | A | - | - | 0.000211 | 0.8561 | 0.1399 | rs429358 | 0.1391 | 0.005 (0.679) |
| LDST/DSST (from previous reports) | *WDR72* | - | 15 | A | G | - | - | 0.157(min P) | -3.6077 | 0.009968 | rs138481566 | 0.009968 |  |

**References:**

Braskie, M., Jahanshad, N., Stein, J., Barysheva, M., Johnson, K., McMahon, K., et al. (2012). Relationship of a variant in the NTRK1 gene to white matter microstructure in young adults. *J Neurosci.*, *32*(17), 5964–72.

Braskie, M., Jahanshad, N., Stein, J., Barysheva, M., McMahon, K., de Zubicaray, G., et al. (2011). Common Alzheimer’s disease risk variant within the CLU gene affects white matter microstructure in young adults. *J Neurosci.*, *31*(18), 6764–70.

Braskie, M., Kohannim, O., Jahanshad, N., Chiang, M., Barysheva, M., Toga, A., et al. (2013). Relation between variants in the neurotrophin receptor gene, NTRK3, and white matter integrity in healthy young adults. *Neuroimage*, *82*(146-53).

Fernandes, C., Westlye, L., Giddaluru, S., Christoforou, A., Kauppi, K., Adolfsson, R., et al. (2014). Lack of association of the rs1344706 ZNF804A variant with cognitive functions and DTI indices of white matter microstructure in two independent healthy populations. *Psychiatry Res.*, *222*(1-2), 60–6.

Hibar D, Stein J, Renteria M, et al (2015) Common genetic variants influence human subcortical brain structures. Nature. doi: 10.1038/nature14101

Ibrahim-Verbaas CA, Bressler J, Debette S, Schuur M, Smith AV, Bis JC D, G, Trompet S, Smith JA, Wolf C, Chibnik LB, Liu Y, Vitart V, Kirin M PK, Polasek O, Zgaga L, Fawns-Ritchie C, Hoffmann P, Karjalainen J LJ, et al (2015) GWAS for executive function and processing speed suggests involvement of the CADM2 gene. Mol Psychiatry doi: 10.10:1–9.

Jahanshad, N., Rajagopalan, P., Hua, X., Hibar, D. P., Nir, T. M., Toga, A. W., et al. (2013). Genome-wide scan of healthy human connectome discovers SPON1 gene variant in fl uencing dementia severity. *Proceedings of the National Academy of Sciences of the United States of America*, *110*(12), 4768 – 4773. doi:10.1073/pnas.1216206110/-/DCSupplemental.www.pnas.org/cgi/doi/10.1073/pnas.1216206110

Jonassen, R., Endestad, T., Neumeister, A., Foss Haug, K., Berg, J., Landrø, N. (2012). The effects of the serotonin transporter polymorphism and age on frontal white matter integrity in healthy adult women. *Front Hum Neurosci*, *6*(19).

Kochunov, P., Glahn, D., Nichols, T., Winkler, A., Hong, E., Holcomb, H., et al. (2011). Genetic analysis of cortical thickness and fractional anisotropy of water diffusion in the brain. *Front Neurosci.*, *doi: 10.33*.

Konrad, A., Vucurevic, G., Musso, F., Stoeter, P., Dahmen, N., Winterer, G. (2009). ErbB4 genotype predicts left frontotemporal structural connectivity in human brain. *Neuropsychopharmacology : Official Publication of the American College of Neuropsychopharmacology*, *34*(3), 641–50. doi:10.1038/npp.2008.112

Lopez, L. M., Bastin, M. E., Maniega, S. M., Penke, L., Davies, G., Christoforou, A., et al. (2012). A genome-wide search for genetic influences and biological pathways related to the brain’s white matter integrity. *Neurobiology of Aging*, *33*(8), 1847.e1–14. doi:10.1016/j.neurobiolaging.2012.02.003

Mounce, J., Luo, L., Caprihan, A., Liu, J., Perrone-Bizzozero, N., Calhoun, V. (2014). Association of GRM3 polymorphism with white matter integrity in schizophrenia. *Schizophr Res.*, *155*(1-3), 8–14.

Nickl-Jockschat, T., Stöcker, T., Krug, A., Markov, V., Maximov, I., Huang, R., et al. (2014). Genetic variation in the G72 gene is associated with increased frontotemporal fiber tract integrity. *Eur Arch Psychiatry Clin Neurosci.*, *Jul 17 201*.

Penke, L., Muñoz Maniega, S., Houlihan, L. M., Murray, C., Gow, A. J., Clayden, J. D., et al. (2010). White matter integrity in the splenium of the corpus callosum is related to successful cognitive aging and partly mediates the protective effect of an ancestral polymorphism in ADRB2. *Behavior Genetics*, *40*(2), 146–56. doi:10.1007/s10519-009-9318-4

Shen, L., Kim, S., Risacher, S., Nho, K., Swaminathan, S., West, J., et al. (2010). Alzheimer’s Disease Neuroimaging Initiative. Whole genome association study of brain-wide imaging phenotypes for identifying quantitative trait loci in MCI and AD: A study of the ADNI cohort. *Neuroimage*, *53*(3), 1051–63.

Sprooten, E., Knowles, E. E., McKay, D. R., Göring, H. H., Curran, J. E., Kent, J. W., et al. (2014). Common genetic variants and gene expression associated with white matter microstructure in the human brain. *NeuroImage*, *97*, 252–261. doi:10.1016/j.neuroimage.2014.04.021

Sprooten, E., Sussmann, J., Moorhead, T., Whalley, H., Ffrench-Constant, C., Blumberg, H., et al. (2011). Association of white matter integrity with genetic variation in an exonic DISC1 SNP. *Mol Psychiatry*, *16*(7), 685, 688–9.

Stein JL, Medland SE, Vasquez AA, et al. (2012) Identification of common variants associated with human hippocampal and intracranial volumes. Nat Genet 44:552–61. doi: 10.1038/ng.2250

Thomason, M., Dougherty, R., Colich, N., Perry, L., Rykhlevskaia, E., Louro, H., et al. (2010). COMT genotype affects prefrontal white matter pathways in children and adolescents. *Neuroimage*, *53*(3), 926–34.

Tost, H., Alam, T., Geramita, M., Rebsch, C., Kolachana, B., Dickinson, D., et al. (2013). Effects of the BDNF Val66Met polymorphism on white matter microstructure in healthy adults. *Neuropsychopharmacology.*, *38*(3), 525–32.

Wang, R., Fratiglioni, L., Laukka, E., Lövdén, M., Kalpouzos, G., Keller, L., et al. (2015). Effects of vascular risk factors and APOE ε4 on white matter integrity and cognitive decline. *Neurology*, *84*(11), 1128–35.
